# Reliable Freestanding Position-Based Routing in Highway Scenarios

**DOI:** 10.3390/s121114262

**Published:** 2012-10-24

**Authors:** Gabriel A. Galaviz-Mosqueda, Raúl Aquino-Santos, Salvador Villarreal-Reyes, Raúl Rivera-Rodríguez, Luis Villaseñor-González, Arthur Edwards

**Affiliations:** 1 Electronics and Telecommunications Department, CICESE Research Center, Ensenada-Tijuana Highway, Km. 3918, Playitas, C. P. 22860, Ensenada, Baja California, Mexico; E-Mails: agalaviz@cicese.edu.mx (G.A.G.-M.); rrivera@cicese.mx (R.R.-R.); 2 Faculty of Telematics, University of Colima, Av. University 333, C. P. 28040, Colima, Col., Mexico; E-Mails: aquinor@ucol.mx (R.A.-S.); arted@ucol.mx (A.E.); 3 Plantronics Inc. Mexico, Av. Producción No. 216 Parque Industrial Internacional Tijuana, Mexico; E-Mail: luis.villasenor@plantronics.com

**Keywords:** geographic routing, VANET, highway, location service

## Abstract

Vehicular *Ad Hoc* Networks (VANETs) are considered by car manufacturers and the research community as the enabling technology to radically improve the safety, efficiency and comfort of everyday driving. However, before VANET technology can fulfill all its expected potential, several difficulties must be addressed. One key issue arising when working with VANETs is the complexity of the networking protocols compared to those used by traditional infrastructure networks. Therefore, proper design of the routing strategy becomes a main issue for the effective deployment of VANETs. In this paper, a reliable freestanding position-based routing algorithm (FPBR) for highway scenarios is proposed. For this scenario, several important issues such as the high mobility of vehicles and the propagation conditions may affect the performance of the routing strategy. These constraints have only been partially addressed in previous proposals. In contrast, the design approach used for developing FPBR considered the constraints imposed by a highway scenario and implements mechanisms to overcome them. FPBR performance is compared to one of the leading protocols for highway scenarios. Performance metrics show that FPBR yields similar results when considering freespace propagation conditions, and outperforms the leading protocol when considering a realistic highway path loss model.

## 1. Introduction

In the context of Intelligent Transport Systems (ITS), Vehicular *Ad Hoc* Networks (VANETs) are considered the key technology required to radically improve safety [1,2]. Their importance can also be inferred from the growing interest that VANETs have drawn from entities directly involved in ITS research and development, [3–5]. In fact, according with the National Highway Traffic Safety Administration, VANETs (also called vehicle-to-vehicle communications or V2V) potentially address 79% of all pre-crash scenarios involving unimpaired drivers [1]. Furthermore, it is also expected that a wide range of applications will become available by enabling V2V communication or vehicle-to-infrastructure communications (V2I). Some examples of important applications include safety [6], Internet connectivity [7], entertainment through multiplayer games [8], file sharing support [9], lane change assistance [2] and traffic control [10].

For several years, consortiums and car manufacturers around the World have focused their efforts on enhancing driver safety, comfort and efficiency [11–16]. These efforts have already provided meaningful results, which range from simulation tools [17] to real prototypes of vehicular safety applications [18]. A significant result is a family of international standards that shares a common architecture, networking protocols and air interface definitions for VANET wireless communications. This family of standards is called Communication Access for Land Mobile (CALM) [19], which was adopted by the European community in 2006. Additionally, the Institute of Electrical and Electronic Engineers (IEEE) has significantly contributed to VANET development by providing a series of standards for Dedicated Short-Range Communications (DSRC), such as the IEEE 802.11p standard [20]. The research community has also been actively contributing in different research areas related to the development of VANETs. Examples of active research topics in VANETs include medium access control methods [21], novel applications [8,9], security [22,23] and routing protocols [24–26].

In VANETs the network topology is *ad hoc* in nature. Hence, no infrastructure beyond the network adapter inside the vehicles is necessary. Importantly, the self-dependence of VANETs offers two main advantages: ubiquitous information sharing and low implementation costs. Although there are several communication standards that can be used to establish a radio link between vehicles in a VANET such as mobile WiMAX [27], IEEE 802.11b radios [28–30] and Bluetooth [31–33], currently the IEEE 802.11p standard is emerging as the most prominent option. The IEEE 802.11p standard is based on the physical layer (PHY) of the IEEE 802.11a standard. The main difference is that IEEE 802.11p limits the channel bandwidth to 10 MHz and sets the operating frequency to 5.9 GHz [34]. Importantly, the Medium Access Control (MAC) of IEEE 802.11p is still based on the Carrier Sense Multiple Access with Collision Avoidance (CSMA/CA) method. Despite the advantages offered by CSMA/CA, a significant issue arising when using this MAC mechanism is that it cannot guarantee message dissemination by itself. Another issue with CSMA/CA is that it cannot provide a time frame for packet delivery because of the exponential back off mechanism. Lastly, it is worth mentioning that IEEE 802.11p adapts the enhanced distributed channel access (EDCA) mechanism of the IEEE 802.11e standard (with some modifications to the transmission parameters). Shortly, the EDCA mechanism defines different distributed coordination function (DCF)-CSMA/CA parameters for four different traffic classes: background, best effort, video and voice, [20,35,36].

Currently, the design of routing strategies represents one of the most important research topics for VANETs. In fact, routing is one of the most critical and challenging components that must be solved to enable V2V communications. For example, the routing protocol must deal with the constraints imposed by the high mobility of vehicles. Furthermore, constrains such as mobility patterns, fading wireless channel, density of vehicles and the availability of infrastructure are closely related to the specific deployment scenario, *i.e.*, highway or urban [26,37]. For example, the speeds achievable on highways are significantly higher than in urban scenarios. Thus, a protocol designed to work in urban scenarios may not be able to cope with the higher vehicle speeds present in highway scenarios. Therefore, the specific application scenario should be considered when designing a routing strategy in order to adequately address the particular constraints imposed by a given scenario. In this context, it is important to note that highways account for a significant amount of the road infrastructure deployed throughout several countries. For example, highways represent about 75% of the total statute miles in the U.S. [1]. Deploying roadside infrastructure to provide coverage for the entire network of roads and highways would take a long time and require a huge investment. Hence, enabling vehicle communications in highways through the use of VANETs is an important scenario that should be further studied, as recently research have clearly pointed out [6,21,38–42].

This paper introduces an efficient multi-hop routing mechanism to enable V2V communications in highway scenarios without employing underlying infrastructure. The routing algorithm was developed considering that the vehicles speed can be as high as 60 m/s under regular traffic conditions. Furthermore, other important constraints pertaining to this application scenario are considered within the routing algorithm design including: dynamic transmission ranges, vehicle acceleration, and high vehicle mobility. Our proposed routing strategy addresses these issues by using a broadcast approach with a light retransmission mechanism in the location service, altogether coupled with a reliable position-based routing algorithm. It is important to note that the routing strategy considers realistic propagation conditions following the results reported in [37]. Our main contributions include:

1. A new location service based on a broadcast approach coupled with a new broadcast suppression technique. The location service addresses the hard conditions imposed by the wireless channel, providing a high success rate for route discoveries.
2. A novel light routing protocol that considers the constraints imposed by a realistic channel propagation model in its design.

The remainder of this paper is organized as follows: Section 2 provides a literature review. Section 3 introduces the Freestanding Position Based Routing (FPBR) protocol proposed in this paper. Section 4 then presents several performance metrics obtained when using FPBR for V2V communications in highway scenarios. The performance evaluation setup is also described in this section. Finally, Section 5 presents the conclusions of this work.

## 2. Related Work

The VANETs routing problem was initially addressed by using several well-known routing protocols such as AODV, DSR, and GPSR [43–45], which were originally developed for mobile *ad hoc* networks (MANETs). However, these protocols were not designed to cope with the specific constraints found in VANETs such as dynamic transmission ranges, vehicle acceleration and high vehicle mobility [46]. Consequently, the performance of these protocols for use in VANETs is not as good as that shown for low mobility applications. Subsequent routing protocols, such as those introduced in [47–50], were specifically developed for VANET applications. In [48], the authors propose a junction-based unicast routing algorithm for urban VANETs called GyTAR. This algorithm consists of three main stages: traffic density estimation, intersection selection and data forwarding between intersections. The features of the GyTAR protocol aim to overcome packet losses. Nevertheless, GyTAR assumes the presence of fixed wireless routers at intersections, which may not be easy to deploy in highway scenarios. Another feature of GyTAR is that it assumes that the vehicles' geographic position is provided by an ideal external location service (LS). The drawback with the ideal LS assumption is that the LS effects in the routing metrics are unknown. For the radio channel, GyTAR considers a radio range, based on the two-ray model. Another junction-based routing algorithm for urban scenarios called CMGR was introduced in [51]. In this work, the authors propose an algorithm to discover a route to any gateway attached to a backhaul network. The geographic greedy forwarding algorithm is used by CMGR to relay the packets between intersections. The discovery service of CMGR is similar to route establishment in AODV. Although the broadcast mechanism implemented in CMGR allows fast packet propagation, CMGR does not consider any redundancy strategy. Including a redundancy strategy within the broadcast mechanism is very important because severe wireless channel conditions can cause significant packet losses. The CMGR and GyTAR protocols addressed important challenges commonly found in VANETs. Nevertheless, it can be argued that their use is not the most adequate for highway scenarios as they are infrastructure dependent [41]. Additionally, as shown in [37], the radio channel in highway scenarios does not correspond to the propagation models used in the performance evaluation of these protocols. Another routing protocol developed for urban scenarios named TO-GO is introduced in [50]. TO-GO is a geo-opportunistic routing protocol that requires previous knowledge of the VANET topology in order to select the next forwarder node. For this purpose, each node in TO-GO must construct a 2-hop neighbors table by means of periodic beacon messages. In addition to the next forwarder node, the transmitter node selects a set of backup nodes by means of a complex procedure involving the use of Bloom filters. The chosen forwarder node and the set of backup nodes form a forwarder set that uses a distance-based timer to determine which node will retransmit the packet first. In order to avoid the broadcast storm problem, every node in the forwarder set must be able to hear each other. However, it is important to take into account that the Bloom filter can give false positives in dynamic data sets [52], thus possibly causing unwanted transmissions. Although [50] reports good performance metrics for TO-GO when considering a non-ideal path loss model, the implemented channel was not explicitly developed for V2V scenarios. Finally, it is worth mentioning that TO-GO does not implement a location discovery service. CAR is a routing protocol developed for city and highway scenarios [47]. It includes its own location service, which is based on the Preferred Group Broadcasting technique (PGB [53]). In PGB, every relay node is registered in the broadcast packet if its velocity vector direction is non-parallel to the velocity vector direction of the previous forwarder node. The location service includes a retransmission mechanism, which consists of retransmitting the broadcast packet if the original sender does not hear the transmission of the same packet from a forward neighbor. Although this retransmission mechanism helps overcome the loss of packets, it could also lead to unnecessary retransmissions. Furthermore, route replay is performed by a unicast strategy, which has one main drawback: other nodes may not be warned about the search. This may lead to unnecessary searches, with the consequent waste of resources. The GPSR-L protocol was introduced in [49] with the aim of overcoming some of the problems shown by GPSR when used in VANETs. In GPSR-L, a lifetime threshold for each link is introduced in the neighbors table in order to overcome the lack of a prediction mechanism in GPSR. With the introduction of this threshold, GPSR-L aims to overcome erroneous next-hop selections caused by old information stored in the neighbors table. Nevertheless, for highway scenarios, erroneous next-hop selections caused by the channel can still occur as a deterministic radio channel was assumed in [49]. The LORA-CBF protocol is a position- based routing algorithm introduced in [54]. This protocol implements a clustering algorithm where only the gateway nodes are allowed to retransmit packets. Nevertheless, since freespace propagation conditions are assumed in LORA-CBF, the clustering algorithm does not guarantee cluster stability in highway scenarios. This may lead to packet losses and/or delays, because it may be difficult to form clusters or an excessive number of clusters may be formed (consisting of a single node) depending on the particular channel conditions at any given moment. Another problem arising with LORA-CBF in highway scenarios is that the most forward within radius neighbor is selected as the next-hop when disseminating data. As it will be explained in the following sections, this assumption may lead to significant packet losses caused by the constraints imposed by the highway V2V channel. In [39], the authors introduced a routing protocol specifically developed for VANETs deployment in highway scenarios. This protocol was named Destination Discovery Oriented Routing (DDOR). Compared to routing protocols previously introduced for VANETs, the DDOR protocol offers advantages such as a built-in location service and a low complexity position prediction algorithm. The inclusion of these mechanisms is important, because they help to overcome erroneous next-hop selections. Additionally, DDOR proposes enabling the IEEE 802.11 RTS/CTS mechanism as a redundancy strategy for its location service and for the data dissemination stages. The LS included in DDOR is an important feature that makes DDOR more suitable for highway scenarios. Nevertheless, one weakness of DDOR is that its redundancy strategy is not controlled by itself. A consequence of this lack of control is that erroneous next-hop selections may lead to a significant overhead increase in the MAC layer. Despite this weakness, it seems that for highway scenarios DDOR overcomes several of the problems shown by the routing protocols previously mentioned. In fact, in [39] several performance metrics were provided for DDOR, showing that this protocol outperforms AODV, DSR and GPSR.

It has been suggested that GPSR-L may offer good performance in highway scenarios compared to CAR, GPSR and GyTAR [26]. However, GPSR-L was not developed with the highway scenario in mind. Furthermore, for this scenario GPSR-L has the drawback that a packet retransmission strategy, a discovery service, and a strategy to handle the acceleration of vehicles were not implemented. The inclusion of these strategies in a routing protocol for highway VANETs is very important, because of the wireless channel constraints imposed by these scenarios may lead to significant packet losses. Furthermore, a self-dependent algorithm is more suitable for this kind of scenarios [26,39]. In contrast, DDOR was explicitly developed for highway scenarios. Therefore, it does include a packet retransmission strategy and a discovery service.

One common characteristic found in AODV, DSR, GPSR, GyTAR, TO-GO, CGMR, LORA-CBF, CAR, GPRS-L and DDOR is that most of them where designed without considering a specific channel model. In fact, most of them have been evaluated by considering an ideal path loss model or channel models that were not specifically developed for V2V communications. Although this assumption is a good starting point for the design and evaluation of a routing strategy, recent works have shown that the path loss model has a significant impact in the routing algorithm performance [55]. Furthermore, the authors in [37] showed that the relative direction of vehicles seriously affects the link path loss in highway scenarios. Therefore, it is really important to consider the path loss model when designing new routing mechanisms for highway scenarios.

Based on the previous discussion, it can be stated that two significant issues that must be addressed when designing new routing protocols for highway VANETs are: the selection of an adequate V2V wireless propagation channel model [37], and the design of a reliable destination discovery service. As explained in Section 3, the FPBR protocol introduced in this paper considers these design constraints.

## 3. Freestanding Position-Based Routing Protocol

As previously explained, the constraints imposed by highway channels must be considered when developing a routing strategy for highway VANETs, this in order to achieve a satisfactory routing performance when deploying VANETs in this kind of scenarios. Therefore, the different modules of the routing protocol (e.g., the beaconing period, the next-hop selection algorithm, the location discovery service, and location prospection) should be designed with the aim to overcome the drawbacks imposed by the highway scenarios. The next subsections introduce the different modules that comprise the Freestanding Position Based Routing (FPBR) protocol proposed in this paper. These modules were specifically developed for highway VANETs. For the development of FPBR it was assumed that the nodes are equipped with digital maps, a global positioning system (GPS), an IEEE 802.11a radio transceiver or an IEEE 802.11p radio transceiver with a single traffic class enabled. It is considered that all the nodes in the VANET use the same radio transceiver (either IEEE 802.11a or IEEE 802.11p). Additionally, a distributed strategy for traffic density estimation like the proposed in [56,57] is also assumed.

### 3.1. Radio Channel

It is well known that in a wireless communication system, the impairments introduced by the channel can adversely affect the transmitted signal. In this context, the path loss and the fading are the most important channel properties to consider when analyzing the performance of a V2V wireless communication system, [37,58,59]. In fact, as the path loss increases the SNR at the receiver decreases, resulting in a degradation of the system performance, [37,58,59]. Most of the research dealing with the design of routing protocols for VANETs assumes that the radio channel follows the behavior of traditional propagation models, e.g., freespace, two rays, Nakagami, *etc.* Nevertheless, as shown in [37], these models do not hold for V2V communications in highway scenarios, because of the antennas height and the vehicle direction have different effects over the path loss [37,55,58,59].

Considering an adequate radio channel model for the development of a routing protocol aimed to V2V applications is very important, as can be inferred from the analysis and results reported in [55]. In fact, the results in [55] show that different radio channel models have different impacts on the routing strategy performance. This is a consequence of the VANETs PHY layer intrinsic features, specifically the low antenna heights and the highly dynamic topology, [55,58–60]. Recently, several channel models for V2V communications have been proposed in the literature, e.g., see [37,61–64] and references within [58,59]. Among these works, [37,62–64] provide path loss models for highway scenarios. On the other hand, [61] provides two different small-scale fading models for two different cases of V2V communications in highways: oncoming vehicles, and vehicles traveling in the same direction at the same speed. The models introduced in [61] have been previously used for the evaluation of the IEEE 802.11p standard, [27]. However, these models are not straightforward applicable to the evaluation and design of routing protocols for VANETs in highway scenarios, since the path loss and the shadowing are not considered. Particularly, the path loss is a critical parameter that should be considered when evaluating VANETs routing protocols, because of the SNR of the received signal at different nodes is dependent on the path loss. Furthermore, [61] does not provide a model for vehicles moving away each other in highways, which is a case occurring in highway VANETs [37].

Among the works that provide path loss characterization for highway scenarios (e.g., [37,62–64]), the model introduced in [37] stands out because it is derived from extensive V2V measurement campaigns. Moreover, this model agrees well with previous findings as those reported in [63]. The path loss model introduced in [37] follows a classic power law and includes a large-scale fading (shadowing) term. Furthermore, the path loss model in [37] allows the characterization of three different V2V communications cases with one single model, specifically: oncoming vehicles, vehicles traveling in convoy, and vehicles travelling away from each other. Therefore, this work considers the highway path loss model introduced in [37] for the development of the routing strategy, since this model was specifically developed for V2V highway scenarios. The path loss model introduced [37] is described by Equation (1):

(1) $$\begin{array}{cc} PL(d)=PL_{0}+10\text{nlo}g_{10}\left(\frac{d}{d_{0}}\right)+X_{\sigma }+\zeta PL_{c} & d>d_{0} \end{array}$$

where *d* is the propagation (*T_x_* − *R_x_*) distance; *PL*_0_ is the path loss at a reference distance *d*_0_; *n* is the path loss exponent; *X_σ_* is a zero-mean normally distributed random variable with standard deviation *σ; PL_c_* is a correction term that accounts for the offset between forward and reverse path loss. An important characteristic of this path loss model is the introduction of the *ζ* variable, which is defined according to the relative direction of vehicles. Specifically, *ζ* is set to: 1 for vehicles travelling in opposite directions and moving away; −1 for vehicles travelling in opposite directions and getting closer; and 0 for vehicles in travelling in convoy. Specific values for every parameter in (1) can be found in [37].

### 3.2. Beacon Rate

The vehicles (nodes) choose the next hop for sending data or control messages based on information previously exchanged with neighbors. This information is exchanged by means of beacon messages. Consequently, there is a tradeoff between the overhead caused by the beacons and the age of the information in the one-hop neighbors table. As different VANET scenarios have different mobility patterns, the strategy to handle this tradeoff depends on the scenario specific characteristics [26]. Thus, setting an adequate beacon rate is an important task, since overly high beacon rates may lead to unnecessary overhead whereas overly low beacon rates may lead to erroneous next hop selections, (e.g., see [65]). Therefore, setting an adequate beacon rate is an important task that depends on the particular routing protocol features. As an example AODV, GyTAR, LORA-CBF and GPSR use a fixed beacon rate of 1 beacon per second, whereas DDOR uses a variable beacon rate ranging from 0.2 to 0.4 beacons per second.

The same set of vehicles is available most of the times in highway scenarios, [26]. Therefore, it may be feasible to use a location prediction (LP) algorithm to achieve a relatively low beacon rate. Consequently, a LP algorithm has been implemented within FPBR in order to predict the gap between the stored and the actual position of neighbor vehicles. This LP algorithm makes possible to achieve beacon rates of 0.4 when considering a free flow vehicle density (this density implies that the vehicles will reach its maximum speed [66,67]), and beacon rates of 0.3 for other vehicle densities.

### 3.3. Next-Hop Selection Algorithm

#### 3.3.1. Location Prediction Algorithm

When a vehicle needs to send or relay a packet, the location prediction algorithm must fill the gap between the last values stored in the one-hop neighbors table and the actual values of each neighbor. Thus, when a next-hop neighbor has to be selected, the LP algorithm is invoked before performing the selection. The LP algorithm implemented in FPBR works as follows:

1. The number of entries in the one-hop neighbors table is updated as follows: if the age of the position information for one particular neighbor is older than 2 times the beacon rate, then this particular neighbor is deleted from the one-hop neighbors table.
2. After performing step 1, the position of all neighbors is updated in the one-hop neighbors table by means of Equation (2):
  (2) $$P_{\text{prospected}}=P_{\text{current}}+(\hat{v}*dt+\hat{\text{acc}}*dt^{2})$$
  where *v̂* is the last stored velocity vector of the neighbor; *acĉ* is the last stored acceleration vector of the neighbor; and *dt* is the information dwell time in the one-hop neighbors table.
3. The updated one-hop neighbors table is passed to the next-hop selection algorithm.

#### 3.3.2. Selection Algorithm

The FPBR protocol selects the next hop node by first predicting the actual position of each node with the LP algorithm. After prediction, vehicles are grouped in three different sets, based on their relative direction to the sender vehicle: vehicles traveling in opposite directions and moving away belong to group *V_s_*; vehicles traveling in opposite directions and approaching belong to group *V_a_*; and vehicles traveling in the same direction belong to group *V_c_*. The final selection is made considering the most forward within adjusted radio (MFWAR) mechanism introduced in this paper. The adjustment of the radio range coverage is performed considering the radio propagation model introduced in [37] as described by Equation (1).

##### The Most Forward Within Adjusted Radius (MFWAR) mechanism

Equation (1) shows that it is very important to consider the relative direction of the neighbors when the next forwarder is selected. According to Equation (1), vehicles travelling in opposite directions and approaching have a higher probability of successfully receiving a packet, because of the relative path loss decrease (*i.e*., *ζ* is set to −1). When vehicles move away from each other in opposite directions, the probability of dropping a packet becomes higher because of the relative path loss increase (*i.e.*, *ζ* is set to 1). If the vehicles are traveling in convoy, then there is neither a relative path loss increase nor a relative path loss decrease (*i.e.*, *ζ* is set to 0). Note that this does not imply that the path loss remains unchanged when the vehicles travel in convoy, since the shadowing effects are still considered by Equation (1). Furthermore, from Equation (1) it can be inferred that considering the transmitter nominal radio coverage for the selection process may not be the best option, since the shadowing may lead to significant packet losses near the border of the nominal transmission range. Thus, in order to decrease the probability of dropping a packet, the most forward within radius (MFR) technique (see [68] for a description of MFR) was modified to include the use of three dynamic factors named *β_a_*, *β_c_,* and *β_s_*. The dynamic factors are used to scale the nominal radio coverage to a more accurate value for each set of vehicles *V_a_*, *V_c_,* and *V_s_* respectively. We call this new dissemination technique *Most Forward Within Adjusted Radius* (MFWAR).

**Figure 1.**
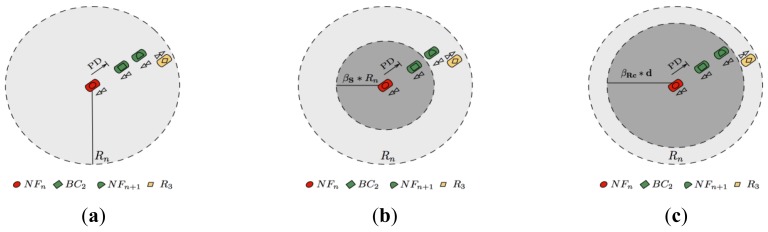
Use of the dynamic factors *β_s_*. and *β_c_* to adjust the nominal radio range, *R_n_*, for vehicles belonging to the *V_s_* and *V_c_* sets. (**a**) Nominal radio coverage *R_n_*; (**b**) Adjusted *β_s_* * *R_n_* radio range for vehicles traveling in opposite direction and moving away (*V_s_* set). (**c**) Adjusted *β_c_* * *R_n_* radio range for vehicles traveling in the same direction (*V_c_* set).

In order to explain how the dynamic factors are used, assume that node *T*_1_ in Figure 1(a) has nominal radio range *R_n_*, and that *β_s_* and *β_c_* are the scaling factors for the *V_s_* and *V_c_* sets respectively. Additionally the following assumptions are made for this figure:

- Node *T*_1_ has data to send beyond its nominal radio coverage.
- Node *R*_3_ moves away from *T*_1_ and thus it belongs to the *V_s_* set.
- Nodes *R*_1_ and *R*_3_ travel in the same direction of *T*_1_ and belong to the *V_c_* set.

If freespace propagation had been considered, then *R*_3_ would have been selected as a relay node. This is because *R*_3_ is within *T*_1_ nominal radio coverage and *R*_3_ is the farthest node (see Figure 1(a)). However, *R*_3_ may not be the best option when considering the highway path loss model described by Equation (1), since *T*_1_ and *R*_3_ are moving in opposite directions and *R*_3_ is near the border. Thus, the MFWAR mechanism adjusts *T*_1_ radio coverage by means of *β_s_* for vehicles belonging to set *V_s_*, as is the case for *R*_3_. Therefore, *T*_1_ radio range is adjusted to the *β_s_* * *R_n_* value and *R*_3_ is not selected because it is out of range (see Figure 1(b)). On the other hand, *R*_1_ and *R*_2_ are vehicles that belong to the *V_c_* set and MFWAR adjusts the radio range for these vehicles to *β_c_* * *R_n_*. Note that*βc* < 1 in order to avoid selecting vehicles near the border which may lead to significant packet losses. Although *R*_1_ and *R*_2_ are within the adjusted *β_c_* * *R_n_* radio range, *R*_2_ is the vehicle most forward (see Figure 1(c)) and therefore it is chosen as a relay node (*FN_n_*_+1_)

### 3.4. Location Service

The FPBR protocol invokes the location service (LS) when a packet arrives for a vehicle with unknown geographic position. The LS is performed with broadcast packets. Therefore, in order to eliminate the broadcast storm problem, a suppression broadcast technique is included in FPBR. Additionally, a redundancy strategy is also introduced in order to improve the LS robustness. The proposed algorithm for the LS is detailed in the next subsections:

#### 3.4.1. Destination Discovery

Three classes of nodes are considered in the destination discovery procedure: source nodes, relay nodes, and destination nodes. The source nodes are vehicles with data to send. The relay nodes are vehicles located along the dissemination path. This nodes relay destination discovery packets. The destination nodes are the vehicles towards which the data packets are sent.

If the geographic position of a destination node is unknown and a source node wants to send data packets to that vehicle, then a location request packet (LREQ) is broadcasted. This packet is used to find the geographic position of the destination node. Before broadcasting the LREQ, a transmitter (*i.e.*, the source node or a relay node) selects a set of vehicles as relay nodes through FPBR's selection algorithm (see Section 3.3.2). If the vehicle is the source node, then the set of relay nodes in the LREQ packet must include at least one vehicle for each direction. The same applies for relay nodes located at intersections. On the other hand, relay nodes not located at intersections select vehicles located towards the dissemination direction before broadcasting the LREQ packet.

When the LREQ packet arrives at the destination node, it waits for twice the propagation time, *τ*, before sending a location reply packet (LREP). This waiting time is introduced in order to avoid possible packet collisions with LREQ packets that could arrive from different paths. Thus, after 2*τ* s the destination node generates the LREP containing its geographic position and speed. The LREP packet is disseminated towards the source node position using the same algorithm as the LREQ. When the LREP packet reaches the source, the data packet is forwarded towards the geographic position of the destination node by means of the MFWAR mechanism. In order to avoid collisions between LS packets and hello beacons, every node that hears a LS packet delays its beacon period by *τ*. Vehicles along the discovery messages (LREP and LREQ) path are aware of these packets since they are broadcast packets.

#### 3.4.2. Redundancy Strategy

**Figure 2.**
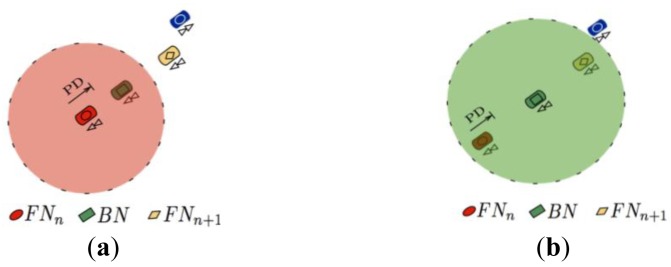
Example of the redundancy strategy implemented in FPBR. (**a**) The discovery packet reaches the *BN* node but it does not reach the *FN_n_*_+1_ node; (**b**) The *BN* node rebroadcasts the discovery packet towards the primary relay node since the original discovery packet did not reach the *FN_n_*_+1_ node.

As previously mentioned, the aim of the MFWAR selection mechanism is to reduce the loss of discovery packets by considering the hard constraints imposed by the radio channel. Nevertheless, sometimes the discovery packets may not reach the intended primary relay node because of larger than expected path losses or inaccurate position predictions made by the selection algorithm (see Figure 2(a)). Therefore, if the discovery packet sent by the current forwarder node does not reach the primary relay node, then the FPBR algorithm will use a redundancy strategy that considers the use of a backup node, *BN*. The task of *BN* is to rebroadcast the discovery packet towards the primary relay node in order to continue with the discovery packet dissemination (see Figure 2(b)). As such, it is important to mention that *BN* does not modify the primary relay nodes table in the discovery packets. Note that the backup node must be located between the current forwarder node (named *FN_n_*) and the primary relay node (named *FN_n_*_+1_) in order to improve the reception probability. Besides choosing a backup node, an acknowledgment mechanism is needed to implement the redundancy strategy. However, in the IEEE 802.11a and IEEE 802.11p MAC layer there is no acknowledgment mechanism for broadcast packets. Therefore, a light acknowledgment mechanism is also included in FPBR. This mechanism and the backup node selection procedure are explained next.

##### Light Acknowledgment Mechanism

**Figure 3.**
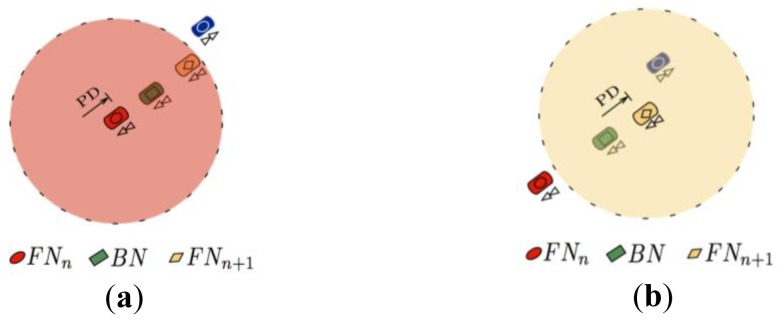
Example of the Light Acknowledgement Mechanism implemented in FPBR. (**a**) The primary relay node successfully receives the discovery packet; (**b**) The *BN* node successfully receives the implicit acknowledgment from *FN_n_*_+1_.

If the primary relay node, *FN_n_*_+1_, receives the discovery packet (see Figure 3(a)), then it will broadcast the discovery packet (with updated primary relay nodes entries) towards a new primary relay node (e.g., *FN_n_*_+2_). As *BN* is located between the current forwarder, *FN_n_*, and the primary relay node, *FN_n_*_+1_, *BN* should be able to receive *FN_n_*_+1_ broadcast transmission (see Figure 3(b)). This feature is used in FBPR as an implicit acknowledgment procedure. Thus, if a node receives a discovery packet where its ID is listed as that belonging to a *BN*, then it will wait for 2τ s. If after this time *BN* does not detect the transmission of a broadcast packet from the *FN_n_*_+1_ node listed on the discovery packet, then it will rebroadcast the original discovery packet towards the *FN_n_*_+1_ node. It is important to note that as an implicit acknowledgment approach is being used in FPBR, additional control packets are not required. Therefore, the FPBR acknowledgment mechanism does not introduce additional overhead.

##### Backup Nodes Selection

As previously mentioned, the *BN* nodes specific task is to rebroadcast the original discovery packet if the *FN_n_*_+1_ node does not perform its corresponding broadcast transmission. In FPBR, *BN* is selected in such way that it is located between *FN_n_* and *FN_n_*_+1_. Although choosing a *BN* right on the middle between the original transmitter and the chosen primary relay node may be a good starting point, such a simple selection mechanism does not consider the vehicles direction and the consequent path loss increase/decrease. Thus, FPBR implements the MFWAR mechanism to select the backup nodes as well. However, for the selection of the *BN* node the nominal radio range is assumed to be equal to the distance between the *FN_n_* and *FN_n_*_+1_. This distance will be referred as *d*(*FN_n_*, *FN_n_*_+1_).

For the *BN* node selection through MFWAR, vehicles are grouped in three sets based on their relative direction to the original forwarder node: *V_Ra_*, *V_Rc_* and *V_Rs_*. Additionally, three dynamic factors *β_Ra_*, *β_Rc_* and *β_Rs_* are considered. These factors are used to adjust *d*(*FN_n_*, *FN_n_*_+1_) by means of MFWAR. If the MFWAR mechanism cannot find a *BN* within the required range, then the current forwarder node is chosen as the *BN* node. As previously mentioned, after the selection of a *BN* node its ID is included in the corresponding table in the discovery packet.

**Figure 4.**
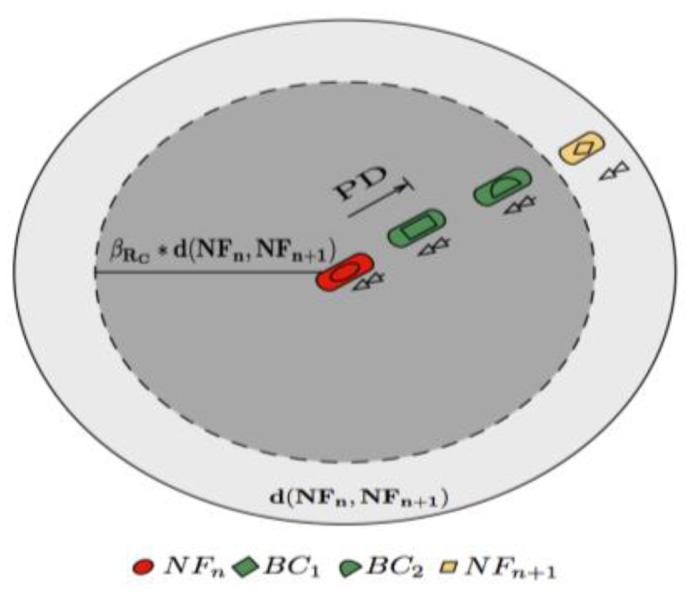
Backup node (*BN*) selection through the MFWAR mechanism. The *BC*_2_ node is chosen as *BN* because it is the nearest to the border of the adjusted distance *β_Rc_* * *d*(*FN_n_*, *FN_n_*_+1_).

An example of the *BN* node selection through the MFWAR mechanism is schematized in Figure 4. Assume that the distance from the original transmitter, *FN_n_*, to the primary relay node, *FN_n_*_+1_, is *d*(*FN_n_*, *FN_n_*_+1_). As both backup nodes candidates in Figure 4 are moving in the same direction as *FN_n_*, *β_Rc_* is used to adjust *d*(*FN_n_*, *FN_n_*_+1_). In Figure 4 the *BC*_2_ node is nearer to the border of the adjusted distance than the *BC*_1_ node. Therefore, *BC*_2_ is chosen as *BN* for this example.

### 3.5. Data Dissemination

After the source node receives the destination geographic position, the data is sent towards the destination using the MFWAR mechanism. It is important to note that as the destination geographic position is included in the data packet header, every relay node can dynamically decide the next hop for the data packet. Furthermore, as every node has a digital map, the anchor-based approach [47,48] can be used where an intersection is found. In FPBR it is assumed that the IEEE 802.11a or the IEEE 802.11p RTS/CTS mechanism is disabled for data dissemination, this assumed to decrease the delay of the data packet and the overhead. Furthermore, implementing the RTS/CTS mechanism requires the transmission of several control packets before sending the actual data. A drawback of this strategy is that one or more of the RTS/CTS control packets could be lost because of the constraints imposed by the highway channel. This may lead to significant performance drops because at least 4 packets (RTS + CTS + DATA + ACK) must be sent to perform a single data packet transmission.

### 3.6. FPBR State Machine and Flow Chart

**Figure 5.**
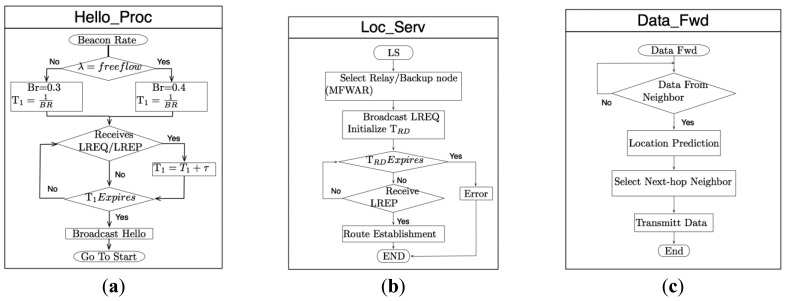
Flow charts for the main processes of the FPBR protocol: (**a**) The Hello mechanism; (**b**) the location service and (**c**) the data dissemination service.

In order to explain the interaction among the different FPBR modules (*i.e.*, Hello_Mechanism, Location Service and Data Forwarding) a flow chart for each one of these modules is depicted in Figure 5. The Figure 5(a) presents the Hello Mechanism flowchart (named Hello Proc module). This module is in charge of sending the hello messages. The waiting period, T1, used in this module is calculated in accordance with the fixed beacon rate mentioned in Section 3.2. The Location Service flowchart is shown in Figure 5(b) (named Loc_Serv). This module is in charge of performing the procedures described in Section 3.4. Lastly, the flowchart for the data forwarding mechanism explained in Section 3.5 is introduced in Figure 5(c) (named Data_Fwd).

**Figure 6.**
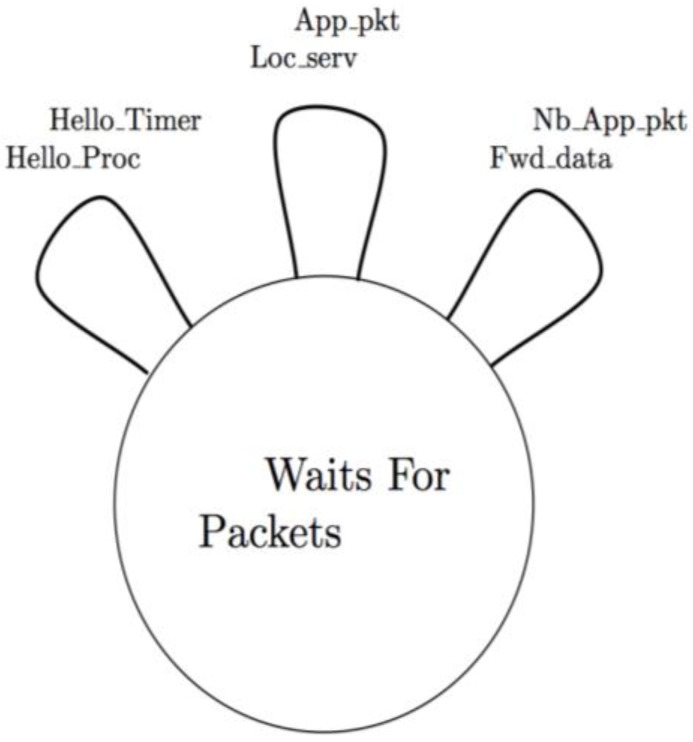
State machine of the interaction between the different modules of FPBR protocol.

The interaction between the different FPBR modules is controlled by means of a state machine (SM), as depicted in Figure 6. Every transition of the SM is in the format condition/procedure. A specific module from Figure 6 is launched when the corresponding condition of the state machine is fulfilled. The Hello timer condition is satisfied when the T1 waiting period ends, then the Hello proc is invoked. If the network layer receives a data packet from the application layer, then the App_pkt condition is fulfilled and the Loc_Serv module is invoked. Finally, if a node receives a data packet sent by a neighbor node, the Nb App_pkt condition is satisfied and the Fwd_Data module is invoked.

## 4. Performance Evaluation

This section introduces several performance metrics obtained when using the FPBR protocol. These performance metrics are compared with those obtained when using the DDOR protocol for benchmark purposes. Particularly, metrics such as packet delivery ratio, delay per hop, overhead, MAC overhead, end-to-end delay and number of hops were obtained for both protocols by means of a simulation setup programmed in the OPNET Modeler simulator. Two different propagation models were considered for the simulation setup: freespace propagation and the highway propagation model introduced in [37] for VANETs. For the simulation setup the IEEE 802.11a OPNET model was modified to resemble the IEEE 802.11p PHY/MAC layers as described below.

### 4.1. Benchmark Routing Protocol

The DDOR protocol has been used in this work as a benchmark for the performance of FPBR. The DDOR protocol was chosen because, as mentioned in the introduction, it was explicitly developed for the deployment of VANETs in highway scenarios. As such, in [39] it was shown that DDOR outperforms AODV, DSR, and GPSR. Furthermore, DDOR includes features specifically designed to deal with some of the impairments imposed by highway scenarios, which are not considered by other V2V protocols such as GPSR-L, LORA-CBF and CAR.

The DDOR protocol includes a discovery service coupled with a packet retransmission strategy. As previously mentioned, including these strategies in a VANET routing protocol for highways is very important, since the wireless channel constraints imposed by these scenarios may lead to significant packet losses. The DDOR protocol proposes enabling the IEEE 802.11 RTS/CTS mechanism for its location service and for the data dissemination stages. Thus, DDOR implements a unicast strategy for its location service. These unicast packets are sent in all directions in order to discover the geographic position of the destination node. Additionally, DDOR proposes an adaptive beacon mechanism (AB) based on the traveled distance of the vehicle. The AB mechanism goal is to reduce the overhead caused by the hello messages. Shortly, the AB mechanism sends a beacon message every time that a vehicle crosses a predefined milestone. Finally, DDOR includes a location prediction algorithm based on the vehicle relative velocity and the size of the transmitted packet. This location prediction algorithm is used before sending any packet in order to select the next-hop vehicle.

In order to compare FPBR and DDOR under the same scenario conditions both protocols were implemented in the OPNET Modeler simulator [69]. The FPBR implementation follows the state machine and flowchart introduced in Section 3.6. The DDOR protocol was implemented following the guidelines provided in [39].

### 4.2. Evaluation Setup

#### 4.2.1. IEEE 802.11a OPNET Model PHY/MAC Adaptation to IEEE 802.11p PHY/MAC Parameters

As previously mentioned, the simulation testbed for FPBR and DDOR was programmed in the OPNET Modeler simulator. This is a well recognized simulation suite widely used in the industry and academia for the evaluation of communication networks, [35,70,71]. However, by the time this research work was completed, a specific model for the IEEE 802.11p standard was not included in the OPNET Modeler (v.16.0). Nevertheless, because of the relevance of the IEEE 802.11p standard within the context of V2V communications, the simulation setup used an adaptation of the IEEE 802.11a model such that all PHY/MAC settings and parameters correspond to those found in the IEEE 802.11p standard. This approach was previously used in [35] for the evaluation of AODV and DSR in VANETs equipped with IEEE 802.11p transceivers.

As mentioned in the introduction, the MAC layer in IEEE 802.11p adopted the IEEE 802.11e EDCA mechanism. This mechanism defines four different classes of traffic with different distributed coordination function (DCF)-CSMA/CA parameters, [20,35,36]. In this paper IEEE 802.11p DCF parameters corresponding to best effort traffic over service channels (see [36]) were used to modify the IEEE 802.11a OPNET model. Therefore, the minimum and maximum contention windows sizes, and the time slot length were adjusted to IEEE 802.11p best effort traffic values. Similarly, the DIFS value was replaced by the corresponding AIFS value. Regarding the PHY layer adaptation, the bandwidth and operating frequency of the IEEE 802.11a OPNET model were adjusted to 10 MHz and 5.880 GHz respectively, as defined for IEEE 802.11p standard. Additionally, the data rates of the IEEE 802.11a OPNET model were adjusted to those defined by the IEEE 802.11p standard. For the rest of this paper the modified model will be referred as adapted IEEE 802.11a/p model.

#### 4.2.2. Simulation Scenario

The performance of both protocols was evaluated considering a highway scenario with two lanes for cars travelling in one direction and other two lanes for cars travelling in the opposite direction. The length of each lane was set to 3 km and the width to 4 m. When a vehicle reaches the end of the road, it is reinserted in the lane with vehicles traveling in the opposite direction. The maximum allowed speed was set to 60 m/s. The radio channel propagation model introduced in [37] was implemented in the simulation setup. Both protocols were tested considering this model which, as previously mentioned, was specifically developed for V2V highway scenarios. Additionally, performance metrics for both protocols were obtained considering the freespace propagation model. The mobility pattern for each vehicle was generated following the intelligent driver model introduced in [66]. This is a popular model used to generate mobility patterns for highway and urban scenarios, (e.g., [50,72]). Furthermore, as the vehicles acceleration is an important parameter that could modify the network topology [21], both protocols were evaluated under various maximum vehicle acceleration rates ranging from *α* = 1.6 m/s^2^ to *α* = 5 m/s^2^. Free flow, medium, high and jam vehicle densities were considered in order to evaluate the effects of the vehicle density, *λ*, in the routing metrics. The specific value of *λ* for each density was taken from [67] as shown in Table 1. The adapted IEEE 802.11a/p model with data rate of 6 Mbps was considered for the vehicle transceiver. The performance metrics for each protocol were obtained considering four transmitters. At the beginning of every simulation trial, every transmitter is set to wait for 5 s before starting any transmission. This 5 s period was set to better allow the exchange of hello messages. When the waiting period concludes each transmitter chooses a random starting time and a random destination node. Once a vehicle starts transmitting, it generates packets for an equivalent user data rate of 2 Kbytes per second. This data rate is maintained during the entire simulation. A minimum of 100 trials were performed for each acceleration-density pair. Each trial lasted 300 s during which different performance metrics were recorded. The particular values of each variable used in the simulation are presented in Table 1.

### 4.3. Metrics

The FPBR and DDOR performance was evaluated using the following metrics:

- Packet delivery ratio (PDR). This metric is measured as the ratio between the number of data packets received by the destination and the number of data packets transmitted by the source.
- Delay per hop. This is the average time a data packet requires for a single hop transmission.
- Average end-to-end delay (EED). The EED is the average time for a data packet to reach its destination.
- Network overhead. This metric is measured in terms of the number of routing control packets transmitted per second normalized by the vehicle density. This metric includes the number of beacons plus the discovery control packets.
- MAC Overhead. This metric is measured in terms of the number of control packets that the MAC layer transmits per second normalized by the vehicle density.
- Number of hops (NH). The NH is the average number of hops required for a data packet to reach its destination.

### 4.4. Results and Analysis

Two different radio propagation models were considered for the evaluation of DDOR and FPBR. Thus, the performance metrics are presented in two subsections, one for each propagation model. The results obtained when assuming freespace propagation are introduced in Subsection 4.4.1. Additionally, Subsection 4.4.2 presents the metrics obtained when considering the radio propagation model explicitly developed for highway scenarios introduced in [37].

#### 4.4.1. Results Considering the Freespace Channel

**Figure 7.**
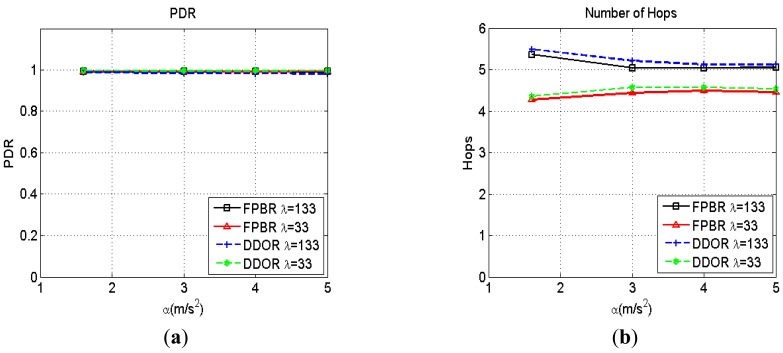
Packet delivery ratio (PDR) and number of hops (NH) obtained with FPBR and DDOR when increasing the maximum acceleration, *α*. Two different vehicle densities were considered for these plots: *λ* = 133 and *λ* = 33. (**a**) PDR *vs. α*; (**b**) Number of Hops *vs. α*.

**Figure 8.**
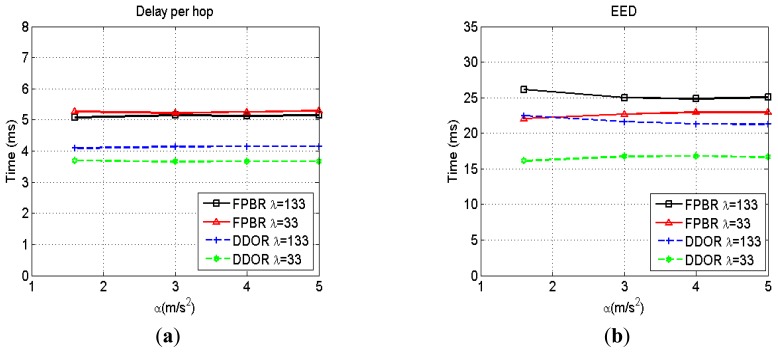
Delay per hop and end-to-end delay (EED) obtained with FPBR and DDOR when increasing the maximum acceleration, *α*. Two different vehicle densities were considered for these plots: *λ* = 133 and *λ* = 33. (**a**) Delay per hop *vs. α*. (**b**) EED *vs. α*.

**Figure 9.**
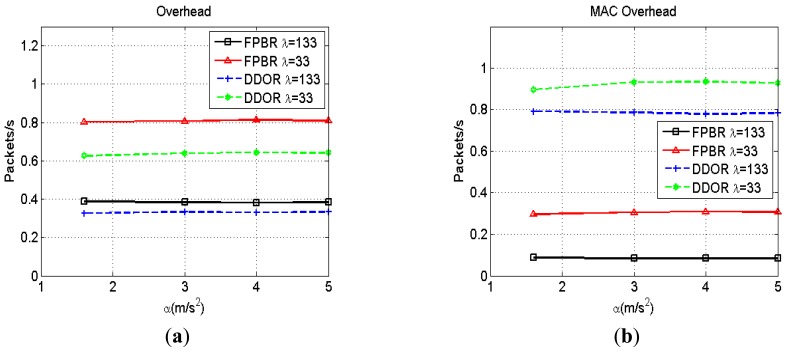
Network and MAC overhead obtained with FPBR and DDOR when increasing the maximum acceleration, *α*. Two different vehicle densities were considered for these plots: *λ* = 133 and *λ* = 33. (**a**) Network overhead *vs.α*. (**b**) MAC overhead *vs. α*.

As discussed in Section 3, the freespace propagation model does not hold for the radio channel conditions present in highway scenarios as measured for V2V communications [37]. However, it is an important scenario commonly used for evaluation purposes [55]. As no fading is introduced when considering the freespace channel, every transmitted packet will reach the transceiver nominal radio range. Thus, for this channel the collision probability for broadcast packets is higher than that observed when considering a fading channel. Therefore, the effect of the packets collisions on the routing strategy performance can be observed with more detail when assuming freespace propagation. The routing metrics obtained when increasing the maximum acceleration range, *α*, for two different densities, *λ*, are shown in Figures 7–9. It can be seen in Figure 7(a) that the packet delivery ratio (PDR) is above 98% for both protocols. Because of the transmitting radio range is deterministic for freespace propagation, any packet loss is caused by collisions or erroneous predictions for both protocols. Thus, as Figure 7(a) shows, both kinds of packet losses are adequately handled by the particular redundancy strategy implemented in DDOR and FPBR. Similar conclusions can be drawn for the number of hops (NH), the end to end delay (EED) and the average delay per hop as shown in Figure 7(b), Figure 8(a,b) respectively. In fact, it can be readily seen in Figures 7 and 8 that both protocols perform similarly well when considering the PDR, NH, EED, and delay per hop metrics.

The network and the MAC overhead metrics are presented in Figure 9. According with Figure 9(a), DDOR generates fewer control packets than FPBR at the network layer. This behavior is observed because DDOR implements its redundancy strategy at the MAC layer by using the RTS/CTS mechanism. On the other hand, Figure 9(b) shows that FPBR yields better MAC overhead metrics than DDOR. This behavior is observed because the RTS/CTS mechanism used in DDOR needs a minimum of three additional packets to transmit a single data packet.

#### 4.4.2. V2V Channel Propagation Model

**Figure 10.**
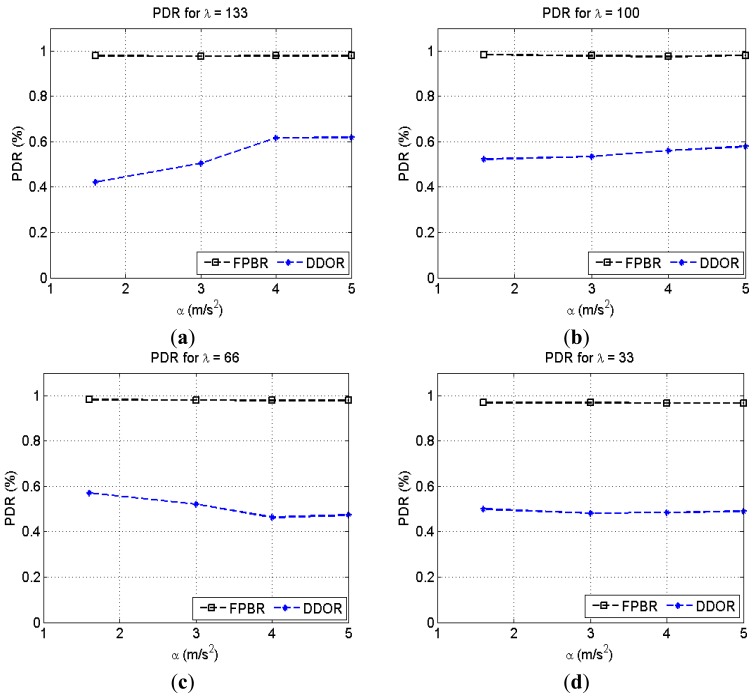
Packet Delivery Ratio (PDR) obtained with FPBR and DDOR when increasing the maximum acceleration, *α*. Four different vehicle densities, *λ*, were considered for these plots. Each plot shows the PDR *vs.* α for: (**a**) *λ* = 133; (**b**) *λ* = 100; (**c**) *λ* = 66 and (**d**) *λ* = 33.

This subsection presents the metrics obtained when considering the radio propagation model explicitly developed for highway scenarios introduced in [37]. Remember that for this channel a transmitted packet may not reach the transceiver nominal radio range because of the path loss and the shadowing (see Section 3.1). Therefore, for this case a packet may not reach its intended destination because of collisions or adverse channel conditions. The Figure 10 presents the PDR obtained with FPBR and DDOR when increasing the maximum vehicle acceleration, *α*. Four different vehicle densities, *λ*, were considered for this figure. Note in Figure 10 that DDOR provides a much lower PDR than that observed when considering freespace propagation. In contrast, the PDR obtained when using FPBR shows a similar performance to that observed in Figure 7(a). This performance drop for DDOR is caused by the underestimation of the V2V highway channel effects when selecting the next forwarder node. As explained in Section 3, FPBR considers the specific constraints imposed by the V2V highway channel when selecting the next forwarder node. Thus, when considering the V2V highway channel FPBR does not show a significant performance drop, compared to the performance achieved when considering the freespace channel. Additionally, note that the PDR is not significantly affected by the acceleration rate when using FPBR. This means that the backup mechanism implemented in FPBR is able to cope with packet collisions and erroneous predictions made by the LP mechanism.

**Figure 11.**
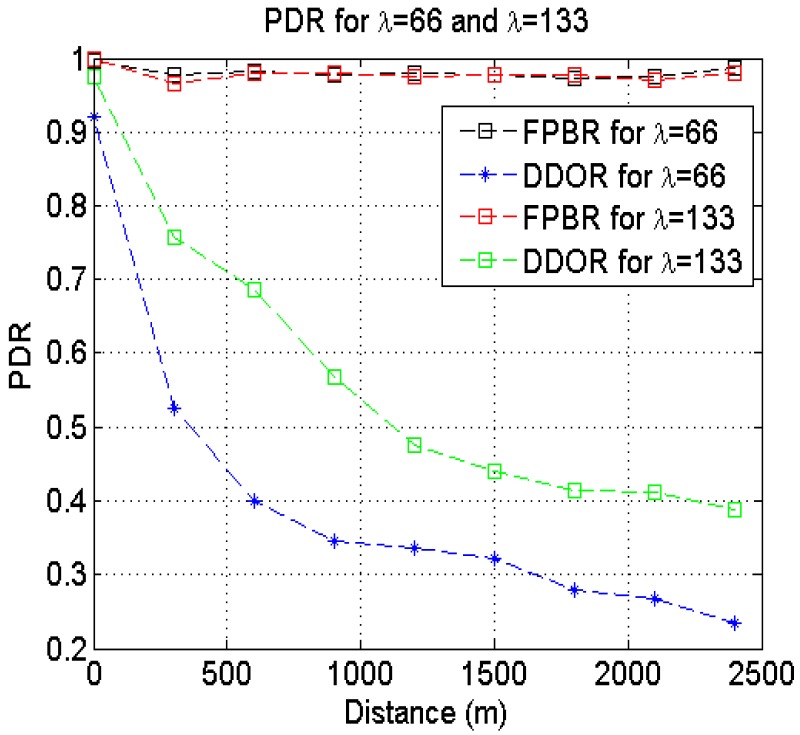
Packet Delivery Ratio (PDR) obtained with FPBR and DDOR when increasing the source-destination distance. The maximum vehicle acceleration was set to *α* = 4. Two different vehicle densities, *λ* = 66 and *λ* = 133, were considered for these figures.

The Figure 11 shows the PDR obtained by FPBR and DDOR for different source-destination distances. The plots in Figure 11 were obtained by setting *α* = 4 with *λ* = 66 and *λ* = 133 Importantly, note in this figure how the PDR obtained with DDOR exhibits a decreasing behavior as the source-destination distance increases. This is caused by erroneous next-hop selections made by DDOR (remember that for this protocol the channel effects are not considered when performing the next-hop selections). In contrast, FPBR provides a significantly higher PDR when compared to DDOR. The FPBR protocol exhibits this behavior because of its backup and next-hop selection mechanisms enable the delivery of packets traveling longer source-destination distances.

**Figure 12.**
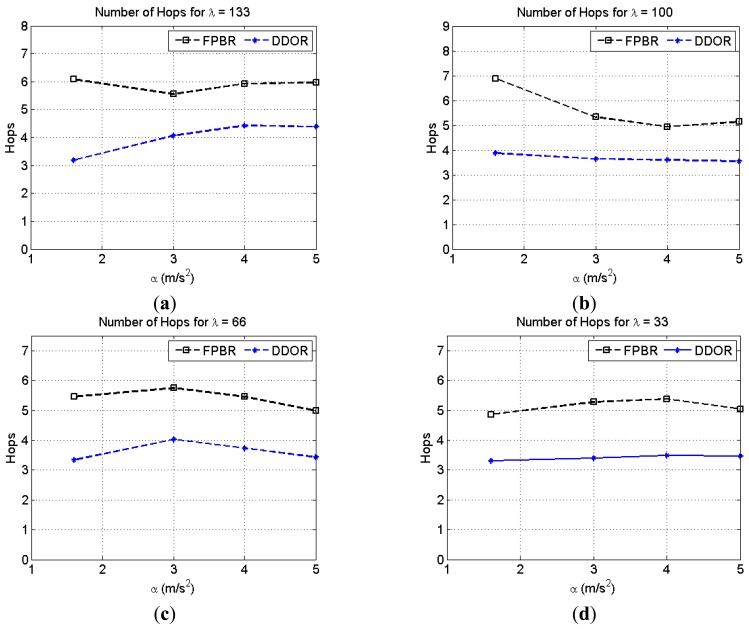
Average number of hops obtained with FPBR and DDOR when increasing the maximum acceleration, *α*. Four different vehicle densities, *λ*, were considered for these plots. Each plot shows the number of hops *vs.* α for: (**a**) *λ* = 133; (**b**) *λ* = 100; (**c**) *λ* = 66 and (**d**) *λ* = 33.

Figure 12 illustrates the effects of increasing the maximum acceleration, *α*, on the average number of hops per packet for different vehicle densities, *λ*. It can be seen in this figure that DDOR has a lower hop count. Although at first this may appear to be an advantage, the reason for the lower hop count in DDOR is the increase in the packet drop probability observed when the source-destination distance is incremented (see Figure 11). On the other hand, the hop count of FPBR is larger because it enables the delivery of packets traveling longer source-destination distances (see Figure 11).

**Figure 13.**
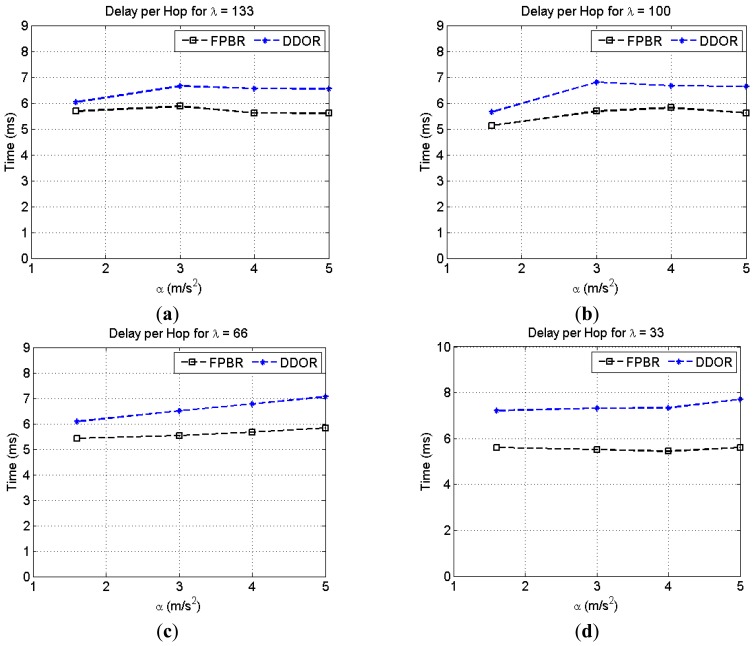
Average delay per hop obtained with FPBR and DDOR when increasing the maximum acceleration, *α*. Four different vehicle densities, *λ*, were considered for these plots. Each plot shows the delay per hop *vs.* α for: (**a**) *λ* = 133; (**b**) *λ* = 100; (**c**) *λ* = 66 and (**d**) *λ* = 33.

Figure 13 shows the effects of increasing the maximum acceleration, *α*, on the average delay per hop metric for different vehicle densities, *λ*. It can be seen that FPBR achieves a lower delay per hop than DDOR. This means that the next hop selection mechanism of FPBR made fewer erroneous selections than those made by the selection algorithm of DDOR. Furthermore, as previously mentioned DDOR's redundancy strategy needs at least 3 control packets while the redundancy strategy of FPBR implements an implicit acknowledgement mechanism. Therefore, when an erroneous selection is made, the redundancy strategy of DDOR generates higher medium access contention than that generated by FPBR's redundancy strategy.

**Figure 14.**
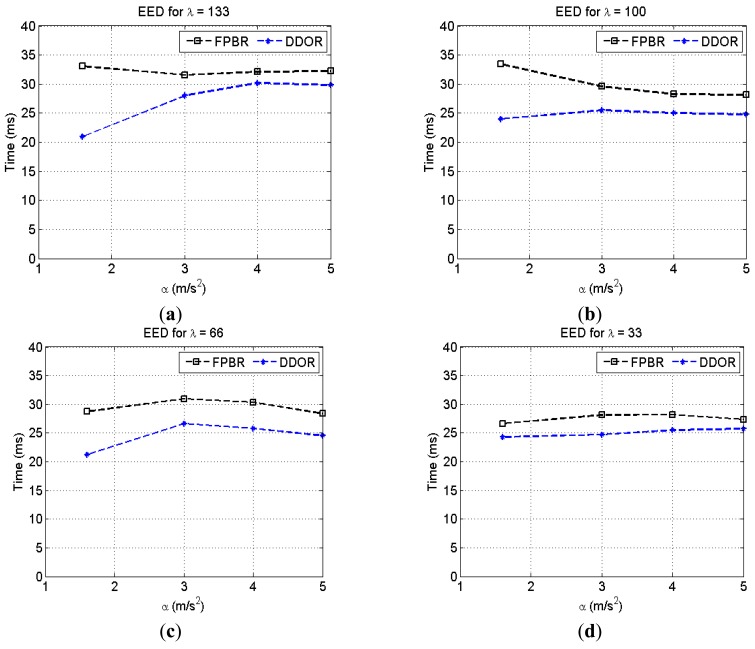
Average end-to-end delay (EED) obtained with FPBR and DDOR when increasing the maximum acceleration, *α*. Four different vehicle densities, *λ*, were considered for these plots. Each plot shows the EED *vs.* α for: (**a**) *λ* = 133; (**b**) *λ* = 100; (**c**) *λ* = 66 and (**d**) *λ* = 33.

Figure 14 illustrates the EED metric behavior when increasing the maximum acceleration, *α*, for different vehicle densities, *λ*. It can be seen in this figure that when using DDOR and increase in *α* leads to an increase in the EED metric for *λ* = 133 and *λ* = 100 (*i.e.*, medium and jam vehicular densities). In contrast, when using FPBR the changes in the acceleration rate do not lead to an increase in the EED for these vehicular densities. The FPBR protocol shows this behavior because of its particular next-hop selection algorithm, described in Section 3.3.2. Thus, it can be inferred that by using the dynamic factors *β_a_*, *β_c_* and *β_s_* the probability of performing an erroneous next hop selection is decreased. Note that for the free flow vehicle density, *λ* = 33, the change in the acceleration, *α*, does not seem to have a significant effect in the EED metric. This behavior is observed because the location prediction mechanism of both protocols is more accurate for this vehicle density.

**Figure 15.**
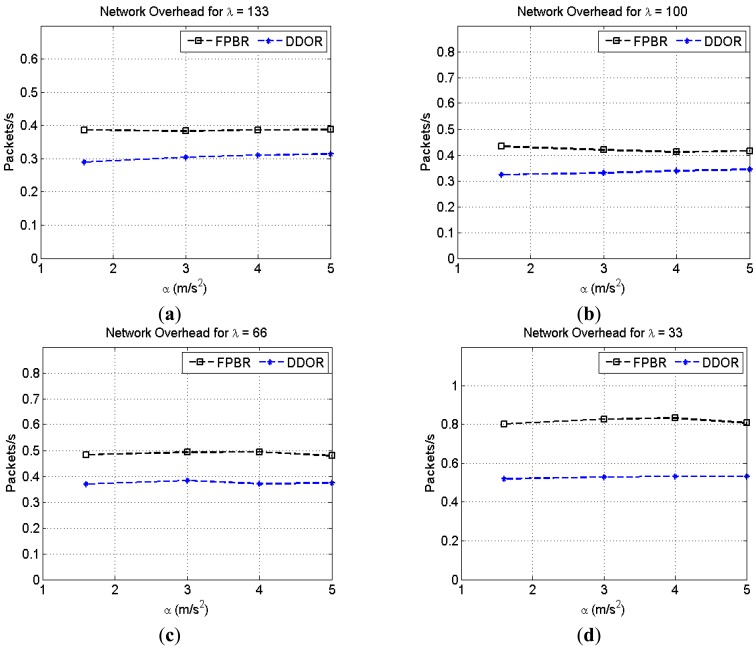
Normalized network overhead obtained with FPBR and DDOR when increasing the maximum acceleration, *α*. Four different vehicle densities, *λ*, were considered for these plots. Each plot shows the network overhead *vs.* α for: (**a**) *λ* = 133; (**b**) *λ* = 100; (**c**) *λ* = 66 and (**d**) *λ* = 33.

Figure 15 shows the effects of increasing the maximum acceleration, *α*, on network overhead metric for different vehicle densities, *λ*. As previously mentioned, the redundancy strategy of DDOR is performed at the MAC layer. Thus, the overhead introduced by DDOR at the network layer mainly consists of hello messages. Consequently, the network overhead for DDOR is lower than that introduced by FPBR (see Figure 15). Note that for *λ* = 33 both protocols show a higher network overhead (see Figure 15(d)). This is caused by the particular adaptive beacon rate mechanisms implemented in both protocols (see Sections 3.2 and 4.1).

**Figure 16.**
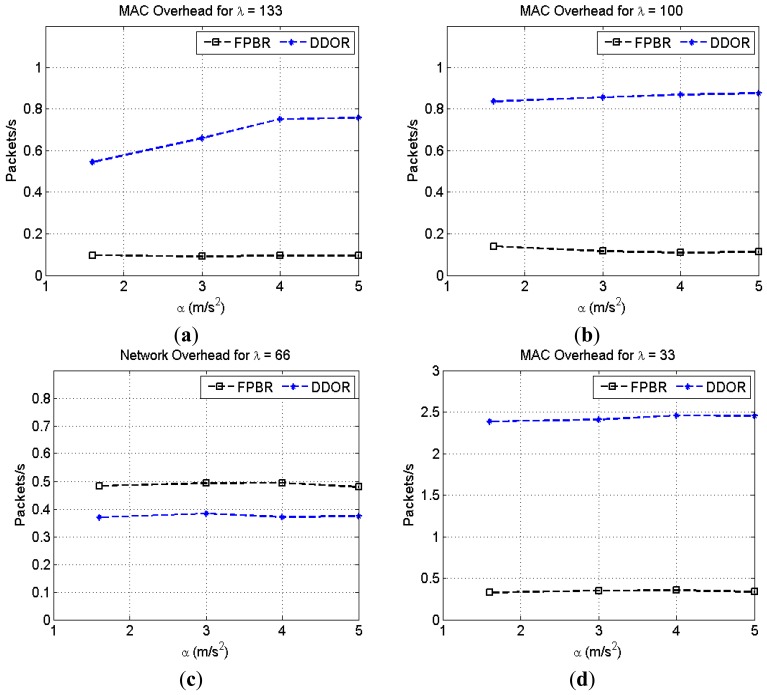
Normalized MAC overhead obtained with FPBR and DDOR when increasing the maximum acceleration, *α*. Four different vehicle densities, *λ*, were considered for these plots. Each plot shows the MAC overhead *vs.* α for: (**a**) *λ* = 133; (**b**) *λ* = 100; (**c**) *λ* = 66 and (**d**) *λ* = 33.

Lastly, the Figure 16 shows the MAC overhead obtained when increasing the maximum acceleration, *α*, for different vehicle densities, *λ*. As mentioned in Section 4.1, the RTS/CTS mechanism used in DDOR needs a minimum of three additional packets to transmit a single data packet. Consequently, this mechanism significantly increases the overhead introduced at the MAC layer. In contrast, FPBR provides a lower MAC overhead. This is because FPBR implements an implicit acknowledgement mechanism in the redundancy strategy for the location service, as explained in Section 3.4. Additionally, FPBR does not enable the RTS/CTS mechanism for the transmission of data packets. Thus, FPBR mitigates the effects of the erroneous selections with fewer packets than those needed by DDOR.

## 5. Conclusions and Future Work

In this paper a new routing protocol for VANETs called Freestanding Position-Based Routing (FPBR) protocol was introduced. The design of FPBR was aimed to the deployment of VANET's in highway scenarios. As such, the performance of FPBR was compared with the performance shown by the DDOR protocol, which is one of the few protocols explicitly developed for V2V communications in highway scenarios. When considering freespace propagation conditions, FPBR delivers performance metrics similar to those obtained when using the leading DDOR protocol. Nevertheless, when considering the V2V highway channel, the performance metrics obtained with both protocols for this scenario show that FPBR outperforms DDOR. The reason for this is because FPBR includes several mechanisms which help to overcome packet losses caused by the harsh conditions imposed by the highway radio channel. Particularly, FPBR's next hop selection and the implicit acknowledgment mechanisms proved to be vital when considering the highway channel, since these routines enabled the selection of suitable primary relay and backup nodes. Consequently, these mechanisms allowed FPBR to yield a high packet delivery ratio. Furthermore, the simulation results show that neither the redundancy strategy nor the location service of FPBR adds a significant overhead, even though the location service uses broadcast packets. Therefore, with the results presented in Section 4.4 it has been shown that in order to improve the performance of the routing protocols for highway scenarios, the constraints imposed by the radio channel must be considered when designing the routing strategy. In that sense, FPBR is a suitable protocol for VANETs deployment in highway scenarios, as it offers several advantages compared to traditional VANETs protocols (e.g., AODV and GPSR-L) and even outperforms the leading DDOR protocol for this kind of scenarios. In this paper the *β* parameters have been chosen based on overall performance for different densities. The performance of FPBR could be improved if the *β* parameters are customized for different network conditions. Thus, future work includes developing an adaptive method to set the *β* parameters values based on current network conditions. In order to adapt FPBR for city scenarios, future work will include obtaining specific *β* parameters for urban scenarios. Regarding the IEEE 802.11p standard, FPBR was evaluated considering one traffic class, delivering satisfactory performance metrics. Similarly, a single traffic class was considered in [35] for the performance analysis of AODV and DSR when using IEEE 802.11p transceivers (as previously mentioned the IEEE 802.11p implementation used in [35] was based in the IEEE 802.11a OPNET model). Therefore, future work will include obtaining performance metrics for FPBR, DDOR, AODV and DSR when considering concurrent traffic flows with different IEEE 802.11p traffic classes.

## Figures and Tables

**Table 1.** Specific values used for each variable in the simulation scenario.

**Parameter**	**Value**
Maximum Velocity	60 m/s
Maximum Acceleration	[1.6–5.0] m/s^2^
Vehicle Density	[33,66,100,133] vehicles/km
Highway Length	3,000 m
Number of Lanes	4
Mobility Model	IDM
Simulation Time	300 s
Packet Size	2 Kbytes
Base Frequency	5.880 GHz
Data Rate	6 Mbps
Transmission Range	300 mts

## References

[1] (2010). Statistics.

[2] Popescu-Zeletin R., Ilja Radusch M.A.R. (2010). Vehicular-2-X Communication: State-Of-The-Art and Research in Mobile Vehicular Ad Hoc Networks.

[3] IEEE. Intelligent Transport Systems Society http://sites.ieee.org/itss/.

[4] ITS-Europe http://ec.europa.eu/transport/its.

[5] Car2Car Communication Consortium http://www.car-to-car.org/index.php?id=46.

[6] Chu Y.C., Huang N.F. (2012). An efficient traffic information forwarding solution for vehicle safety communications on highways. IEEE Trans. Intell. Transp. Syst..

[7] Benslimane A., Barghi S., Assi C. (2011). Fast track article: An efficient routing protocol for connecting vehicular networks to the Internet. Pervasive Mob. Comput..

[8] Tonguz O.K., Boban M. (2010). Multiplayer games over vehicular *ad hoc* networks: A new application. Ad Hoc Netw..

[9] Isento J., Dias J., Neves J., Soares V., Rodrigues J., Nogueira A., Salvador P. FTP@VDTN: A File Transfer Application for Vehicular Delay-Tolerant Networks.

[10] Milanes V., Villagra J., Godoy J., Simo J., Perez J., Onieva E. (2012). An intelligent V2I-based traffic management system. IEEE Trans. Intell. Transp. Syst..

[11] Festag A., Noecker G., Strassberger M., Lubke A., Bochow B., Torrent-Moreno M., Schnaufer S., Eigner R., Catrinescu C., Kunisch J. NoW—Network on Wheels: Project Objectives, Technology and Achievements.

[12] Hartenstein H., Bochow B., Ebner A., Lott M., Radimirsch M., Vollmer D. Position-Aware *Ad Hoc* Wireless Networks for Inter-Vehicle Communications: The Fleetnet Project.

[13] Cooperative-Vehicle-Infrastructure-Systems http://www.cvisproject.org.

[14] Safespot http://www.safespot-eu.org.

[15] Operative Systems for Intelligent Road Safety, C http://www.coopers-ip.eu.

[16] eCoMove http://www.ecomove-project.eu.

[17] Barr R. (2004). An Efficient, Unifying Approach to Simulation Using Virtual Machines. Ph.D. Thesis.

[18] SOUNDBITES: Vehicle-To-Vehicle Communications. http://media.ford.com/article_display.cfm?article_id=33971.

[19] Kosch T., Kulp I., Bechler M., Strassberger M., Weyl B., Lasowski R. (2009). Communication architecture for cooperative systems in Europe. IEEE Commun. Mag..

[20] IEEE Standard for Information Technology (2007). Part 11: Wireless LAN Medium Access Control (MAC) and Physical Layer (PHY) Specifications: Amendment 6: Wireless Access in Vehicular Environments.

[21] Abdel Hafeez K., Zhao L., Liao Z., Ma B. (2011). Clustering and OFDMA-based MAC protocol (COMAC) for vehicular *ad hoc* networks. EURASIP J. Wireless Commun. Netw..

[22] Abumansoor O., Boukerche A. (2012). A secure cooperative approach for nonline-of-sight location verification in VANET. IEEE Trans. Veh. Tech..

[23] Lu R., Li X., Luan T., Liang X., Shen X. (2012). Pseudonym changing at social spots: An effective strategy for location privacy in VANETs. IEEE Trans. Veh. Tech..

[24] Bernsen J., Manivannan D. (2009). Unicast routing protocols for vehicular ad hoc networks: A critical comparison and classification. Pervasive Mobile Comput..

[25] Taysi Z., Yavuz A. (2012). Routing protocols for GeoNet: A survey. IEEE Trans. Intell. Transp. Syst..

[26] Fonseca A., Vazao T. (2012). Applicability of position-based routing for VANET in highways and urban environment. J. Netw. Comput. Appl..

[27] Fernndez-Carams T., Gonzlez-Lpez M., Castedo L. (2011). Mobile WiMAX for vehicular applications: Performance evaluation and comparison against IEEE 802.11p/a. Comput. Netw..

[28] Mizutani K., Kohno R. (2001). Inter-vehicle spread spectrum communication and ranging system with concatenated EOE sequence. IEEE Trans. Intell. Transp. Syst..

[29] Inoue T., Nakata H., Itami M., Itoh K. An Analysis of Incident Information Transmission Performance Using an IVC System That Assigns PN Codes to the Locations on the Road.

[30] Nagaosa T., Hasegawa T. An autonomous Distributed Inter-Vehicle Communication Network Using Multicode Sense CDMA.

[31] Sawant H., Tan J., Yang Q., Wang Q. Using Bluetooth and Sensor Networks for Intelligent Transportation Systems.

[32] Pasolini G., Verdone R. Bluetooth for ITS?.

[33] Sugiura A., Dermawan C. (2005). In traffic jam IVC-RVC system for ITS using Bluetooth. IEEE Trans. Intell. Transp. Syst..

[34] Fernandez J., Borries K., Cheng L., Kumar B., Stancil D., Bai F. (2012). Performance of the 802.11p physical layer in vehicle-to-vehicle environments. IEEE Trans. Veh. Tech..

[35] Iqbal M., Wang F., Xu X., Eljack S., Mohammad A. (2011). Reactive routing evaluation using modified 802.11a with realistic vehicular mobility. Ann. Telecommun..

[36] Mišić J., Badawy G., Mišić V. (2011). Performance characterization for IEEE 802.11p network with single channel devices. IEEE Trans. Veh. Tech..

[37] Karedal J., Czink N., Paier A., Tufvesson F., Molisch A. (2011). Path loss modeling for vehicle-to- vehicle communications. IEEE Trans. Veh. Tech..

[38] Baccelli E., Jacquet P., Mans B., Rodolakis G. (2012). Highway vehicular delay tolerant networks: Information propagation speed properties. IEEE Trans. Inform. Theor..

[39] Sahu P., Wu E., Sahoo J., Gerla M. DDOR: Destination discovery oriented routing in highway/freeway VANET+. Telecommun. Syst..

[40] Rawashdeh Z., Mahmud S. (2012). A novel algorithm to form stable clusters in vehicular *ad hoc* networks on highways. EURASIP J. Wireless Commun. Netw..

[41] Vinel A. (2012). 3GPP LTE *versus* IEEE 802.11p/WAVE: Which technology is able to support cooperative vehicular safety applications?. IEEE Wireless Commun. Lett..

[42] Yan Z., Jiang H., Shen Z., Chang Y., Huang L. (2012). k-Connectivity analysis of one-dimensional linear VANETs. IEEE Trans. Veh. Tech..

[43] Abedi O., Fathy M., Taghiloo J. Enhancing AODV Routing Protocol Using Mobility Parameters in VANET.

[44] Johnson D.B., Maltz D.A., Broch J. (2001). DSR: The dynamic source routing protocol for multi-hop wireless *ad hoc* networks. Ad Hoc Networking.

[45] Karp B., Kung H.T. GPSR: Greedy Perimeter Stateless Routing for Wireless Networks.

[46] Chaurasia B., Tomar R., Verma S., Tomar G. Suitability of MANET Routing Protocols for Vehicular *Ad Hoc* Networks.

[47] Naumov V., Gross T. Connectivity-Aware Routing (CAR) in Vehicular *Ad-Hoc* Networks.

[48] Jerbi M., Senouci S.M., Rasheed T., Ghamri-Doudane Y. (2009). Towards efficient geographic routing in urban vehicular networks. IEEE Trans. Veh. Tech..

[49] Rao S., Pai M., Boussedjra M., Mouzna J. GPSR-L: Greedy Perimeter Stateless Routing with Lifetime for VANETS.

[50] Lee K., Lee U., Gerla M. TO-GO: TOpology-Assist Geo-Opportunistic Routing in Urban Vehicular Grids.

[51] Shafiee K., Leung V.C. (2011). Connectivity-aware minimum-delay geographic routing with vehicle tracking in VANETs. Ad Hoc Netw..

[52] Guo D., Liu Y., Li X., Yang P. (2010). False negative problem of counting bloom filter. IEEE Trans. Knowl. Data Eng..

[53] Naumov V. An Evaluation of Inter-Vehicle *Ad Hoc* Networks Based on Realistic Vehicular Traces.

[54] Santos R., Alvarez O., Edwards A. Performance Evaluation of Two Location-Based Routing Protocols in Vehicular *Ad-Hoc* Networks.

[55] Gozalvez J., Sepulcre M., Bauza R. (2010). Impact of the radio channel modelling on the performance of VANET communication protocols. Telecommun. Syst..

[56] Garelli L., Casetti C., Chiasserini C.F., Fiore M. MobSampling: V2V Communications for Traffic Density Estimation.

[57] Panichpapiboon S., Pattara-atikom W. Evaluation of a Neighbor-Based Vehicle Density Estimation Scheme.

[58] Molisch A., Tufvesson F., Karedal J., Mecklenbrauker C. (2009). A survey on vehicle-to-vehicle propagation channels. IEEE Wireless Commun..

[59] Mecklenbrauker C., Molisch A., Karedal J., Tufvesson F., Paier A., Bernado L., Zemen T., Klemp O., Czink N. (2011). Vehicular channel characterization and its implications for wireless system design and performance. Proc. IEEE.

[60] Wang C., Cheng X., Laurenson D. (2009). Vehicle-to-vehicle channel modeling and measurements: Recent advances and future challenges. IEEE Commun. Mag..

[61] Acosta-Marum G., Ingram M. Six Time- and Frequency-Selective Empirical Channel Models for Vehicular Wireless LANs.

[62] Cheng L., Henty B., Bai F., Stancil D. Highway and Rural Propagation Channel Modeling for Vehicle-to-Vehicle Communications at 5.9 GHz.

[63] Kunisch J., Pamp J. Wideband Car-to-Car Radio Channel Measurements and Model at 5.9 GHz.

[64] Paier A., Karedal J., Czink N., Dumard C., Zemen T., Tufvesson F., Molisch A., Mecklenbruker C. (2009). Characterization of vehicle-to-vehicle radio channels from measurements at 5.2GHz. Wireless Pers. Commun..

[65] Zrar Ghafoor K., AbuBakar K., van Eenennaam M., Khokhar R., Gonzalez A. (2011). A fuzzy logic approach to beaconing for vehicular *ad hoc* networks. Telecommun. Syst..

[66] Treiber M., Hennecke A., Helbing D. (2000). Congested traffic states in empirical observations and microscopic simulations. Phys. Rev. E.

[67] Wang H., Ni D., Chen Q.Y., Li J. (2011). Stochastic modeling of the equilibrium speeddensity relationship. J. Adv. Transp..

[68] Behnad A., Nader-Esfahani S. (2011). On the statistics of MFR routing in one-dimensional *ad hoc* networks. IEEE Trans. Veh. Tech..

[69] Opnet Modeler 16.0 http://www.opnet.com/solutions/network_rd/modeler.html.

[70] Okamura T., Ideguchi T., Tian X., Okuda T. Traffic Evaluation of Group Communication Mechanism among Vehicles.

[71] Opnet Contributed Papers https://enterprise1.opnet.com/tsts/4dcgi/BiblioSearchSubmit?QueryBiblio_whatFindAll&QueryRecordsPerPage1500.

[72] Dressler F., Sommer C., Eckhoff D., Tonguz O. (2011). Toward Realistic Simulation of Intervehicle Communication. IEEE Veh. Tech. Mag..

